# Soft Sensing of Silicon Content via Bagging Local Semi-Supervised Models

**DOI:** 10.3390/s19173814

**Published:** 2019-09-03

**Authors:** Xing He, Jun Ji, Kaixin Liu, Zengliang Gao, Yi Liu

**Affiliations:** 1 Institute of Process Equipment and Control Engineering, Zhejiang University of Technology, Hangzhou 310023, China (X.H.) (K.L.) (Z.G.); 2 College of Computer Science and Technology, Qingdao University, Qingdao 266071, China

**Keywords:** soft sensor, silicon content, semi-supervised learning, extreme learning machine, just-in-time-learning

## Abstract

The silicon content in industrial blast furnaces is difficult to measure directly online. Traditional soft sensors do not efficiently utilize useful information hidden in process variables. In this work, bagging local semi-supervised models (BLSM) for online silicon content prediction are proposed. They integrate the bagging strategy, the just-in-time-learning manner, and the semi-supervised extreme learning machine into a unified soft sensing framework. With the online semi-supervised learning method, the valuable information hidden in unlabeled data can be explored and absorbed into the prediction model. The application results to an industrial blast furnace show that BLSM has better prediction performance compared with other supervised soft sensors.

## 1. Introduction

As a type of metallurgical furnace, blast furnaces are used for smelting to produce industrial metals. The silicon content in hot metal, both as a quality factor and as a chief indicator of the thermal level of the blast furnace, is of central importance. However, it is difficult to measure it online. Additionally, chemical reactions and transfer phenomena in blast furnaces are very complex. Till now, a reliable first-principles model for industrial practice is not available [1,2,3,4,5]. Because of the complexity of the task, the prediction of the silicon content is tricky and it recently attracted much attention. In past two decades, several data-driven soft sensors were proposed to predict the silicon content [6,7,8,9,10,11,12,13,14,15,16,17,18,19,20,21,22,23,24]. For example, some neural networks (NNs) [6,7,8,9,10] and support vector regression (SVR) [17,18] were built as a black-box predictor. The partial least squares regression [11], fuzzy logic approach [12], nonlinear time series analysis [14,15,16], chaos-based iterated multistep predictor [19], multiscale modeling methods [20], and multiple models [21], were also applied to predict the silicon content. Saxén et al. gave a review on data-driven discrete time models for the hot metal silicon content prediction in the blast furnace [22]. These empirical data-driven soft sensor models can be built quickly using the available measured variables [25,26,27,28,29,30,31,32].

In industrial processes, a large amount of sensor variables that are available can be used as input to the soft sensor model. The quality-relevant variable to be predicted using a soft sensor can be regarded as “labeled” data. However, the amount of quality-relevant variable (“labeled” data) is often limited mainly because it is difficult to measure online. Till now, most soft sensors in industrial ironmaking processes act in a supervised manner. That is, for construction of a soft sensor, both of inputs (sensor variables) and outputs (quality-relevant variables) are required for the task of supervised modeling. The labeled dataset contains both input and output data, while the unlabeled one consists of only input data (i.e., large amount of sensor variables). Actually, the labeled data are much fewer than the unlabeled data mainly because the assaying process of silicon contents is infrequent and time-consuming. In contrast, the process input variables are measured frequently. Using a limited set of labeled data, the soft sensors are often inaccurate. To enhance the prediction performance, with large amounts of unlabeled data available, some semi-supervised soft sensors have been applied to chemical processes [33,34,35]. Therefore, the information hidden in unlabeled data is explored to develop a semi-supervised soft sensor for the silicon content prediction.

Most soft sensors have their fixed prediction domains. The predictive accuracy of soft sensors gradually decreases due to changes in the state of chemical plants [36]. Consequently, flexible models with adaptive structure, e.g., just-in-time-learning (JITL) soft sensors [23,24,37] are more attractive than only using a fixed one in practice use. Unfortunately, most conventional JITL-based soft sensors were constructed only with the labeled data. Only the labeled data are considered in the process of selection and modeling of similar samples. Consequently, without integration of useful information in the unlabeled data, the prediction performance of JITL-based models may still not be sufficient for some applications.

In this work, bagging local semi-supervised models (BLSM) for online silicon content prediction are proposed. It integrates the bagging strategy, the JITL modeling manner [37] and semi-supervised extreme learning machine (SELM) [34,38,39] into a unified soft sensing framework. For online prediction of a test sample, the useful information in both of similar labeled and unlabeled samples is taken into its special JITL model. Additionally, a simple bagging strategy is adopted to online construct the model. Compared with conventional JITL models only with the labeled data, the prediction performance of BLSM is improved by utilizing the useful information in unlabeled data.

This work is organized in the following way. The extreme learning machine (ELM) and SELM soft sensors are described in Section 2. Additionally, the BLSM online modeling method and its detailed implementation are proposed in this section. In Section 3, BLSM is applied to online silicon content prediction and compared with other approaches. Finally, a conclusion is given in Section 4.

## 2. Soft Sensor Modeling Methods

In this section, three soft sensing methods for the silicon content prediction are presented. First, the ELM-based supervised regression algorithm is briefly described. Second, the SELM-based semi-supervised regression algorithm is presented. Finally, the BLSM online local modeling method is proposed.

### 2.1. Extreme Learning Machine (ELM) Regression Method

The labeled dataset is denoted as {S}={X,Y}, where $\left\{X^{l}\right\}={\left\{x_{i}^{l}\right\}}_{i=1}^{L}$ and $\left\{Y^{l}\right\}={\left\{y_{i}^{l}\right\}}_{i=1}^{L}$ are *L* input and output data, respectively. ELM works for generalized single-hidden layer feedforward networks (SLFNs) [38]. The ELM model has an input layer, a single-hidden layer, and an output layer. With *N* hidden nodes, ELM approximates the training data, i.e., $\sum_{i=1}^{L}\left\|y_{i}^{l}-{\hat{y}}_{i}^{l}\right\|=0$, where $y_{i}^{l}$ and ${\hat{y}}_{i}^{l}$ denote the actual output and predicted one, respectively. Compactly, the ELM-based regression formulation [38] is described as:

(1) $$P\alpha =Y^{l}$$

where the output matrix of hidden-layer $P={\left[p_{1},p_{2},\ldots ,p_{N}\right]}_{L\times N}$ with $p_{i}={\left[\begin{array}{c} v\left(\langle a_{i},x_{1}^{l}\rangle +b_{i}\right)\\ \vdots \\ v\left(\langle a_{i},x_{L}^{l}\rangle +b_{i}\right) \end{array}\right]}_{L\times 1},i=1,\ldots ,N$; $v\left(\langle a_{i},x_{j}^{l}\rangle +b_{i}\right)$ is the activation function output of the *i*th hidden node related to the *j*th input $x_{j}^{l}$. For the *i*th hidden node, $a_{i}$ and $b_{i}$ are its input weight and bias, respectively; and $\langle a_{i},x_{j}^{l}\rangle$ is the inner product of $a_{i}$ and $x_{j}^{l}$. Here, the commonly used nonlinear sigmoidal function $v\left(x\right)=\frac{1}{1+\exp\left(-x\right)}$ is utilized. The output weights are $\alpha ={\left[\begin{array}{c} \alpha_{1}^{}\\ \vdots \\ \alpha_{N}^{} \end{array}\right]}_{N\times 1}$.

Different from the gradient-descent based training algorithms (e.g. backpropagation method) for many NNs and the optimization method based for support vector machines, the essence of ELM is that the hidden layer of SLFNs need not be tuned. Without resorting to some complex training algorithms, the weights of the hidden neurons in ELM can be efficiently computed [38]. For many regression cases, the number of hidden nodes is much less than the number of training samples, i.e., N<<L. In such a situation, the output weights α [38] are determined as:

(2) $$\alpha ={\left(P_{}^{T}P_{}\right)}^{-1}P_{}^{T}Y^{l}$$

Using the Moore–Penrose generalized inverse of matrix **P** to solve α in ELM is feasible, i.e., $\alpha =P_{}^{+}Y^{l}$ [38]. Additionally, to avoid the problem of $P_{}^{T}P$ being noninvertible, a regularized ELM (RELM) model was formulated [34]

(3) $$\alpha ={\left(P_{}^{T}P+\gamma I\right)}^{-1}P_{}^{T}Y^{l}$$

where γ>0 is the ridge parameter for the unit matrix I. 

Finally, for a test sample $x_{t}={\left[x_{t1},x_{t2},\cdots ,x_{tn}\right]}^{T}\in R^{n}$, its prediction ${\hat{y}}_{t}$ is obtained below:

(4) $${\hat{y}}_{t}=p_{t}\alpha =p_{t}{\left(P_{}^{T}P+\gamma I\right)}^{-1}P_{}^{T}Y^{l}$$

where $p_{t}$ is the output vector of the hidden-layer associated with $x_{t}$.

### 2.2. Semi-supervised Extreme Learning Machine (SELM) Regression Method

For the semi-supervised learning methods, the input and output samples are represented as $\left\{X\right\}=\left\{X^{l}\cup X^{u}\right\}$ and $Y=\left[\begin{array}{l} Y^{l}\\ Y^{u} \end{array}\right]={\left[\frac{\begin{array}{c} y_{1}^{l}\\ \vdots \\ y_{L}^{l} \end{array}}{\begin{array}{l} 0\\ \vdots \\ 0 \end{array}}\right]}_{(L+U)\times 1}$, respectively. Additionally, the hidden layer output matrix P can be defined as $P={\left[p_{1},p_{2},\cdots ,p_{N}\right]}_{\left(L+U\right)\times N}$ as aforementioned. The manifold regularization framework is utilized to learn the matrix W of an SELM model [39].

(5) $$\underset{W}{\min}\;\;\frac{1}{2}\left\{{\left\|JPW-Y\right\|}^{2}+\lambda {\left(PW\right)}^{T}LPW\right\}$$

where ${\left\|JPW-Y\right\|}^{2}$ is the approximation errors of labeled training data (i.e., for the empirical risk) while $\lambda {\left(PW\right)}^{T}LPW$ is the penalty term utilizing the graph Laplacian L with a parameter λ≥0 (i.e., for the complexity of learnt function). All the unlabeled data are integrated into the matrix **P**. The graph Laplacian L can be designed using a basic identity in the spectral graph theory [39]. Additionally, for the convenience of calculation, $J={\left[\begin{array}{l} I_{L}\;\;\;0\\ 0\;\;\;\;0 \end{array}\right]}_{\left(L+U\right)\times \left(L+U\right)}$ is defined [39].

By solving Equation (5), the coefficient matrix W [39] is obtained as:

(6) $$W={\left[\left(J+\lambda L^{T}\right)P\right]}^{+}JY$$

Generally, for semi-supervised learning methods, there is an assumption that the input patterns from both labeled and unlabeled data are from the same distribution. In such a situation, the data samples in the local region should have similar labels [33,34,39]. Useful information hidden in the unlabeled data can be explored from the above modeling framework. The graph Laplacian L of SELM contains the information in both of labeled and unlabeled data. Once the unlabeled data are ignored (i.e., λ=0), W is the same as α in Equation (3). The prediction performance improvement can be obtained by suitably choosing λ as the penalty of model complexity. Finally, for a query sample $x_{t}={\left[x_{t1},x_{t2},\ldots ,x_{tn}\right]}^{T}\in R^{n}$, its prediction ${\hat{y}}_{t}$ is obtained below:

(7) $${\hat{y}}_{t}=p_{t}W=p_{t}{\left[\left(J+\lambda L^{T}\right)P\right]}^{+}JY$$

where $p_{t}$ is the output vector of the hidden-layer associated with $x_{t}$.

### 2.3. Bagging Local Semi-supervised Models (BLSM) Online Modeling Method

In industrial processes, JITL-based local soft sensors are more flexible than only using a fixed one for the relatively long-term utilization [23,24]. Nevertheless, most conventional JITL approaches only use limited labeled data, regardless of the useful information in lots of unlabeled data samples. As can be expected, using the unlabeled data, the prediction accuracy of JITL models can be improved.

Online inquiry of **x***_t_* contains three main steps. First, select a similar set $\left\{S_{t}\right\}=\left\{S_{t}^{l}\cup S_{t}^{u}\right\}$, including both of *L_t_* labeled data and *U_t_* unlabeled data (i.e., $\left\{S_{t}^{l}\right\}=\left\{X_{t}^{l},Y_{t}^{l}\right\}$ and $\left\{S_{t}^{u}\right\}=\left\{X_{t}^{u}\right\}$), from the historical database {S} via some defined similarity criteria [37]. The common Euclidean distance-based similarity is adopted here. Other similarity criteria available [23,24,37] can also be combined with local SELM models. Second, construct a local SELM model *f*(**x***_t_*) using the selected similar dataset $\left\{S_{t}\right\}$. Third, online predict and then repeat the same procedure for another query sample.

For a selected $\left\{S_{t}\right\}$, two parameters, i.e., the number of hidden nodes *N* and the balance parameter λ≥0, are necessary to train a local SELM model. To avoid the overfitting problem, a simple bagging strategy is adopted to generate multiple local candidate models with diversities and then aggregate them as a new predictor. With the bootstrapping re-sampled strategy, several candidate regression models are ensembling to achieve an improved prediction [40].

For the similar labeled dataset $\left\{S_{t}^{l}\right\}=\left\{X_{t}^{l},Y_{t}^{l}\right\}$, *L_t_* pairs of samples are randomly selected to replace $\left\{S_{t}^{l}\right\}$ where the probability of each pair being chosen is $\frac{1}{L_{t}}$ [40]. These *L_t_* pairs of data are a re-sampled training set $\left\{S_{t}^{l}\right\}$. Sequentially, the procedure is repeated for *K* times and to obtain *K* re-sampled datasets, i.e., $\left\{S_{t1}^{l},\cdots ,S_{tK}^{l}\right\}$. Similarly, the bagging strategy is applied to the unlabeled dataset $\left\{S_{t}^{u}\right\}=\left\{X_{t}^{u}\right\}$ to get *K* re-sampled datasets $\left\{S_{t1}^{u},\ldots ,S_{tK}^{u}\right\}$.

For the *k*th dataset $\left\{S_{tk}\right\}=\left\{S_{tk}^{l}\cup S_{tk}^{u}\right\}$, $W_{k}$ of the *k*th local SELM model is obtained (similar with Equations (5) and (6)). Consequently, for a test input $x_{t}={\left[x_{t1},x_{t2},\ldots ,x_{tn}\right]}^{T}\in R^{n}$, the prediction value of the *k*th local SELM model, i.e., ${\hat{y}}_{k,t}$, is formulated:

(8) $${\hat{y}}_{k,t}=p_{t}W_{k}$$

where $p_{t}$ is the output matrix of the hidden-layer associated with $x_{t}$.

Finally, using a simple ensemble strategy, *K* candidate SELM models are equally weighted to generate the final prediction.

(9) $${\hat{y}}_{t}=\frac{1}{K}\sum_{k=1}^{K}{\hat{y}}_{k,t}$$

**Figure 1:**
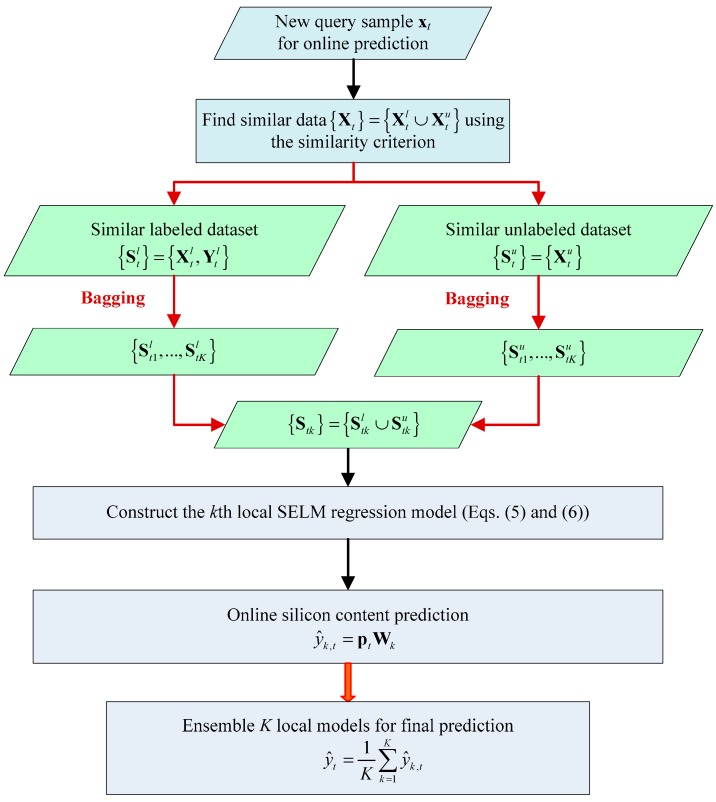
Bagging local semi-supervised models (BLSM)-based online soft sensing flowchart for the silicon content prediction.

The main modeling flowchart of BLSM is given in Figure 1. In summary, BLSM has two main characteristics. First, the useful information hidden in unlabeled data is explored and absorbed. Second, using the bagging strategy [40], the BLSM model can be aggregated using multiple local candidates with diversities.

## 3. Industrial Silicon Content Online Prediction

### 3.1. Data Sets and Pretreatment

The BLSM method is applied to the silicon content prediction in an industrial blast furnace in China. For construction of soft sensors, the related input variables include the blast volume, the blast temperature, the top pressure, the gas permeability, the top temperature, the ore/coke ratio, and the pulverized coal injection rate [22,23,24]. After preprocessing the data set with 3-sigma criterion, most of obvious outliers were removed out. A set of about 260 labeled samples was investigated. Half of labeled samples are considered as the historical samples. The remaining part is used for testing the models. Additionally, 500 unlabeled data were obtained as historical samples in the same furnace. The labeled and unlabeled data are from the same industrial blast furnace, indicating that they share with similar characteristics in a production process. Consequently, the semi-supervised learning methods can be applied.

As a recent supervised method with good nonlinear regression performance, the just-in-time least squares SVR (JLSSVR) soft sensor [23] is adopted for comparison. Additionally, as a semi-supervised model, the SELM model [39] is also combined with JITL to construct a local SELM soft sensor here. Two common performance indices, including the root-mean-square error (RMSE), the relative RMSE (simply denoted as RE), and the hit rate (HR), are adopted and defined, respectively.

(10) $$\mathrm{RMSE}=\sqrt{\sum_{t=1}^{N_{\mathrm{tst}}}{\left(\frac{y_{t}-{\hat{y}}_{t}}{N_{\mathrm{tst}}}\right)}^{2}}$$

(11) $$\mathrm{RE}=\sqrt{\frac{1}{N_{\mathrm{tst}}}\sum_{t=1}^{N_{\mathrm{tst}}}{\left(\frac{y_{t}-{\hat{y}}_{t}}{y_{t}}\right)}^{2}}$$

(12) $$\mathrm{HR}=\frac{\sum_{t=1}^{N_{\mathrm{tst}}}H_{t}}{N_{\mathrm{tst}}}\times 100\%$$

where $N_{\mathrm{tst}}$ is the number of test samples. $H_{t}$ is defined as:

(13) $$H_{t}=\left\{ \begin{array}{l} 1,\;\;\;\left|{\hat{y}}_{t}-y_{t}\right|<0.1\\ 0,\;\;\;\mathrm{else} \end{array}\right.$$

### 3.2. Results and Discussion

**Figure 2:**
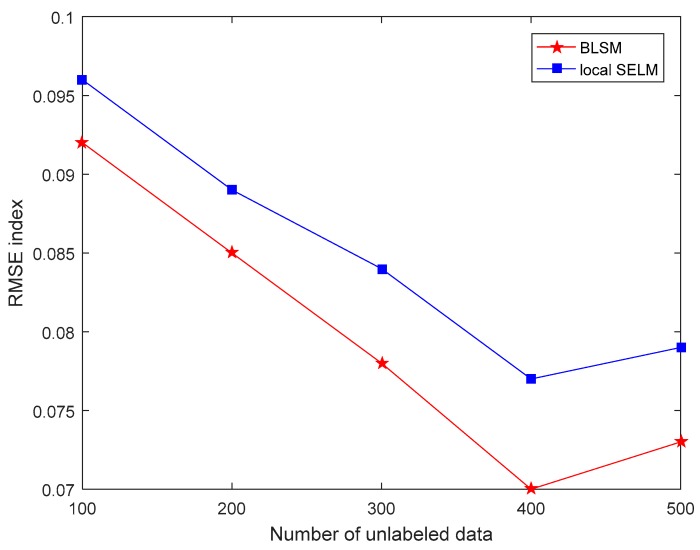
Root mean square error (RMSE) comparison of the silicon content prediction between bagging local semi-supervised models (BLSM) and local semi-supervised extreme learning machine (SELM) models with different numbers of unlabeled data.

**Figure 3:**
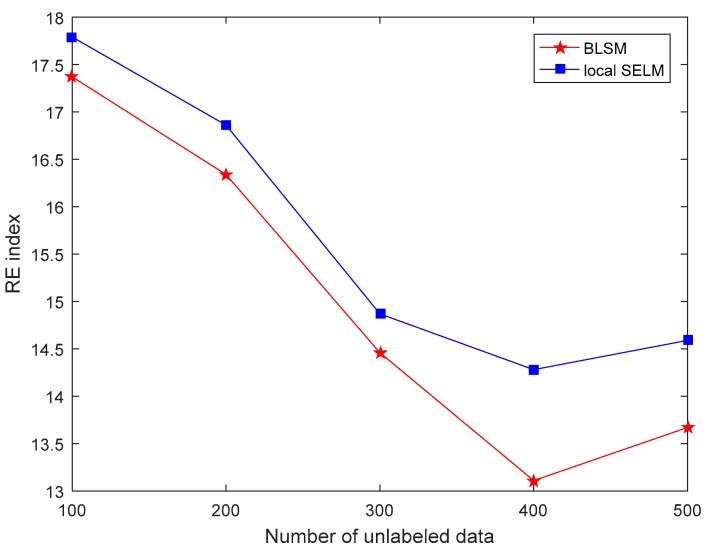
Relative RMSE (RE) comparison of the silicon content prediction between bagging local semi-supervised models (BLSM) and local semi-supervised extreme learning machine (SELM) models with different numbers of unlabeled data.

**Figure 4:**
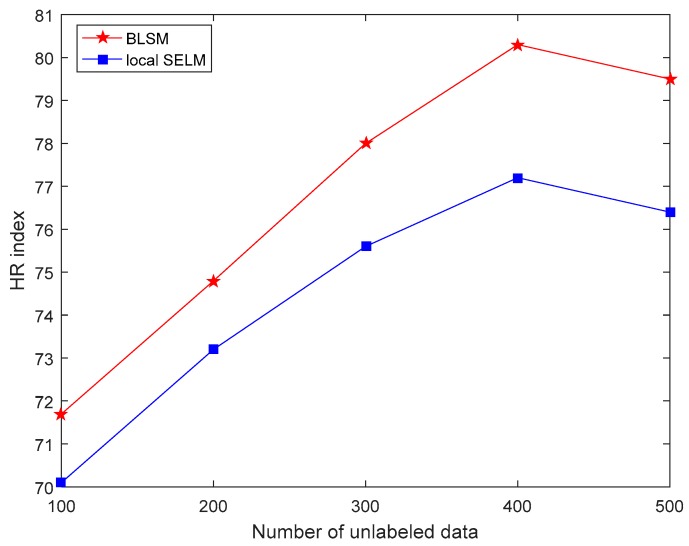
Hit Rate (HR) comparison of the silicon content prediction between bagging local semi-supervised models (BLSM) and local semi-supervised extreme learning machine (SELM) models with different numbers of unlabeled data.

First, with different sizes of unlabeled data, the comparison results of three performance indices between two semi-supervised models, i.e., BLSM and local SELM, are shown in Figure 2, Figure 3 and Figure 4, respectively. For both BLSM and local SELM models, the prediction performance is enhanced gradually with the increase in the size of the unlabeled data. Due to the ensemble local modeling ability, BLSM exhibits superior prediction performance to a single local SELM one. In this case, the prediction performance is not further enhanced when the number of unlabeled samples is more than about 400. This is mainly because most of useful information in unlabeled dataset is absorbed from the first 400 data.

**Figure 5:**
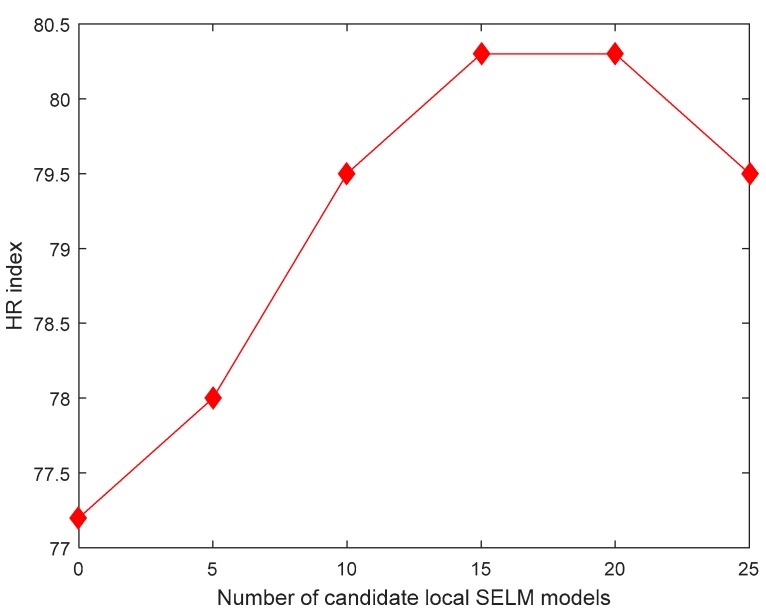
HR comparison of bagging local semi-supervised model (BLSM) different numbers of candidate local semi-supervised extreme learning machine (SELM) models.

With 400 unlabeled data, taking the HR index as an example, different numbers (i.e., *K*) of candidate local SELM models for construction of a BLSM one is shown in Figure 5. With the ensemble learning strategy, the efforts on parameter selection of BLSM can be reduced. The HR index indicates that the ensemble learning can enhance the prediction performance to some extent (the HR value increases from 77.2% to 80.3%). And BLSM achieves the best prediction performance when K=15 for this application. 

**Figure 6:**
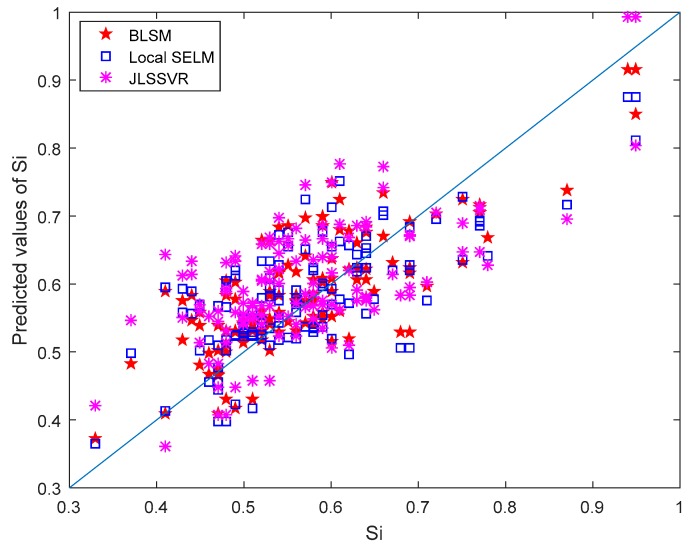
The silicon content assay values against prediction results using bagging local semi-supervised model (BLSM), semi-supervised extreme learning machine (SELM), and just-in-time least squares support vector regression (JLSSVR) soft sensors.

For the three soft sensors (i.e., BLSM, local SELM, and JLSSVR [23]), the silicon content prediction results are shown in Figure 6. This parity plot shows that BLSM is better than local SELM and JLSSVR methods. The prediction performance comparison of three modeling methods is listed in Table 1. Their main characteristics are also described briefly. Generally, BLSM is a local semi-supervised learning model and therefore it can better capture nonlinear characteristics in local regions, especially with the help of unlabeled data. For JLSSVR [23] only with a few labeled data, the prediction domain may be limited. Different from JLSSVR [23], BLSM explores and utilizes the hidden information in lots of unlabeled data to improve the local modeling ability. Moreover, using the simple bagging ensemble strategy, the prediction performance of a semi-supervised local model (e.g., a local SELM) can be enhanced.

The computational complexity of BLSM is about *K* times of a local SELM model. Based on the experiences, *K* is often much less than 100. The online prediction time of BLSM for a test sample is about 1 s (with CPU main frequency 2.3 GHz and 4 GB memory). Compared with the interval time of lab assay, the computational load is accepted. With more historical data (especially unlabeled data), the computational load of online modeling becomes larger. To alleviate this problem, it is suggested that the online and offline models are integrated using the Bayesian analysis [37]. Alternatively, development of the recursive version of BLSM may be a choice. In summary, all the obtained results show that BLSM is a promising prediction method of the silicon content in hot metal produced in blast furnaces.

## 4. Conclusions

This work has presented an online semi-supervised soft sensor model, i.e., BLSM, for blast furnace hot metal silicon content prediction. Two main advantages distinguish BLSM from most current hot metal silicon prediction soft sensors. First, the useful information in unlabeled data is absorbed into the online modeling and prediction framework efficiently. Second, a bagging-based ensemble strategy is integrated into the online semi-supervised model to improve its prediction reliability. The application results show that BLSM has better prediction performance than traditional soft sensors. This is the first application of semi-supervised learning methods to industrial blast furnaces. How to efficiently select the more informative unlabeled data in an error-in-variables environment for construction of a more robust semi-supervised model will be tackled in our future work.

## Figures and Tables

**Table 1:** Detailed prediction performance comparison of semi-supervised and supervised learning models (best results are bold and underlined).

Soft Sensor Models	Brief Description	RMSE	RE (%)	HR (%)
BLSM	Bagging local semi-supervised learning method with ensemble learning strategy	0.070	13.11	80.3
Local SELM	Local semi-supervised learning method without ensemble learning strategy	0.077	14.28	77.2
JLSSVR [23]	Local supervised learning method	0.091	17.43	70.9

## Nomenclature

BLSM	bagging local semi-supervised model
ELM	extreme learning machine
JITL	just-in-time-learning
JLSSVR	just-in-time least squares support vector regression
NNs	neural networks
RE	relative root-mean-square error
RMSE	root-mean-square error
RELM	regularized extreme learning machine
SELM	semi-supervised extreme learning machine
SLFNs	single-hidden layer feedforward networks
SVR	support vector regression
